# Molecular Identification and Benzimidazole Resistance Analysis of Cyathostomins in Chinese Grazing Horses

**DOI:** 10.3390/vetsci13020169

**Published:** 2026-02-09

**Authors:** Chenxue Zhang, Enjia Cai, Yuhui Ma, Guangzhi Zhong, Yu Gao, Yuhong Wu, Bo Liu, Jing Li

**Affiliations:** 1 College of Veterinary Medicine, China Agricultural University, Beijing 100193, China; sy20243051232@cau.edu.cn (C.Z.); 2017305010305@cau.edu.cn (E.C.); sy20233051123@cau.edu.cn (G.Z.); gaoyu@cau.edu.cn (Y.G.); 2 Xinjiang Zhaosu County Xiyu Horse Industry Co., Ltd., Zhaosu, Yili 835600, China; mayuhuim@126.com; 3 Dongfangmadu Equine Teaching Hospital, College of Veterinary Medicine, China Agricultural University, Beijing 100193, China; 4 Beijing Zhongnongda Veterinary Hospital Co., Ltd., Beijing 100193, China; 5 State Key Laboratory of Veterinary Public Health and Safety, Beijing 100193, China

**Keywords:** horses, cyathostomins, anthelmintic resistance, China

## Abstract

This study investigated the cyathostomin species composition, anthelmintic efficacy, and potential resistance-associated mutations in Chinese grazing horses. Fecal samples were collected from 90 adult horses. Fecal egg counts (FECs) were determined using a modified McMaster method. Third stage larvae (L3) cultured from the eggs were identified to species level by PCR amplification and sequencing of the internal transcribed spacer-2 (ITS-2). The main species included *Cylicocyclus nassatus* and *Cylicostephanus* spp. However, differences in the relative abundance of less common species, including *Cyathostomum pateratum* and *Cylicocyclus ashworthi*, suggests regional variation. Anthelmintic efficacy was assessed by a fecal egg count reduction test (FECRT) following albendazole administration. Mutations at six codons of the β-tubulin iso-type-1 (tbb-iso-1) gene were screened by gene sequencing. The results showed that, despite harboring an abundant and diverse cyathostomin community, this herd remained susceptible to albendazole. Overall, this study provides baseline data on cyathostomin species composition and anthelmintic susceptibility in horses from China, contributing to global surveillance of equine cyathostomin resistance.

## Simple Summary

Cyathostomins are the most prevalent intestinal parasites of horses, commonly associated with ill-thrift and reduced performance. This study assessed parasite burdens and species composition in 90 adult grazing horses in China. Fecal egg counts revealed that 88.9% of horses were moderate-to-high egg shedders, indicating substantial environmental parasite pressure. Species identification showed dominance of *Cylicocyclus nassatus* and *Cylicostephanus* spp., consistent with global patterns. All identified cyathostomins remained susceptible to albendazole, and the β-tubulin isotype-1 mutation associated with benzimidazole resistance was not detected. These findings provide important baseline information on cyathostomin infections and anthelmintic resistance in horses from China where such data have been limited, and they may help guide future parasite control strategies.

## 1. Introduction

Small strongyles or cyathostomins are ubiquitous in grazing horses worldwide [1,2]. While horses may tolerate low-level cyathostomin infections asymptomatically, high parasitic burdens are frequently associated with ill-thrift, weight loss, and diminished athletic performance. In some cases, infections progress to acute larval cyathostominosis (ALC). Affected animals typically present with diarrhea, dehydration, hypoalbuminemia, and neutrophilia. ALC has been reported to have a fatality rate of 50% in a retrospective clinical case series in the United Kingdom. A total of 38 clinical cases of ALC were recorded between 2009 and 2020, of which 21 (55%) cases did not survive [3]. Cyathostomins infections are common in grazing horses in China. A 2018 study conducted in Xinjiang—the province with the largest equine population in the country—reported a prevalence rate of 98.11% [4].

Currently, there are over 50 species of cyathostomins, and the species composition appears to vary by region and age group [5]. A recent study has found the existence of several cryptic cyathostomin species, demonstrating the need for continuing research on defining the cyathostomins communities [6]. The characterization of the cyathostomin community can be achieved by morphological identification, reverse line blots and internal transcribed spacer 2 (ITS-2) sequencing [7]. Currently, the species composition of the cyathostomin burden of grazing horses in China remains unclear. Investigating the cyathostomin communities can provide essential baseline data for the species composition and phylogenetic relationships of equine cyathostomins.

Benzimidazoles (BZ) are widely used for the control of nematodes in horses. Their mode of action involves binding to β-tubulin and disrupting microtubule formation, ultimately impairing cellular energy metabolism in the parasite [6,7,8]. It has been reported that the intensive and prolonged use of BZs has driven the emergence of anthelmintic resistance (AR) in equine cyathostomins worldwide [9]. Accordingly, routine monitoring of drug efficacy is now recognized as a critical component of equine health management and parasite-control programs. The fecal egg reduction test (FECRT) is considered the gold standard for describing the AR phenotype [10]. Allele-specific PCR can be used to identify the BZ resistance genotype of equine cyathostomins, based on the detection of single-nucleotide polymorphisms (SNPs) in the *tbb-iso-1*. SNPs in the *tbb-iso-1* at codons 200 and 167 have been reported in equine cyathostomins [11]. Some studies have reported that mutations at codon 200 in BZ-resistant cyathostomins frequently appear as GAG/A, although this nucleotide substitution does not result in an amino acid change [12]. While in the strongylid of ruminants, BZ resistance is predominantly associated with SNPs in codon 167 (F167Y, TTC→TAC) [13], codon 198 (E198A, GAA→GCA), and codon 200 (F200Y, TTC→TAC) [14]. Research on the BZ-resistant cyathostomins genotype in equine remains very limited.

Therefore, to explore the AR status of equine cyathostomins in Chinese grazing horses, this study investigated a horse population that had undergone routine anthelmintic treatment for three consecutive years. This study characterized the species composition of the cyathostomin community, evaluated the efficacy of albendazole in this herd, and screened for cyathostomins that carried β-tubulin mutations associated with BZ resistance. Together, these data provide the first integrated assessment of cyathostomin species diversity and benzimidazole susceptibility in grazing horses from China, offering essential baseline information for improving parasite-control strategies in the region.

## 2. Materials and Methods

### 2.1. Sample Collection

The study was conducted from February 2024 to February 2025 in Xinjiang, China. Fresh fecal samples were collected from 90 adult Ili horses from local grazing herds that had not received anthelmintic treatment within the preceding five months. These horses grazed on grassland during summer and were housed in stables during the winter. Over the past ten years, the horses were routinely dewormed with albendazole.

### 2.2. Fecal Egg Count Test

FECs were performed using the modified McMaster technique, following AAEP recommendations [12]. A multiplication factor of 25 was achieved by mixing 4 g of feces with 26 mL of saturated sodium chloride solution.

### 2.3. L3 Culture and DNA Extraction

Fresh fecal samples containing a minimum of 200 EPG strongylid eggs were cultured at 20 °C under humid conditions for up to 21 days. L3 of cyathostomins were collected from day 14 onward using the Baermann technique and stored at 4 °C [15]. L3 suspensions were examined and cyathostomin L3 was identified based on morphological characteristics, particularly the number of intestinal cells. After identification, 0.05% of sodium hypochlorite (Shijiazhuang Hengjieli Chemical Co., Ltd., Shijiazhuang, China) was used to remove the sheath of L3 [11]. Individual L3’s genomic DNA was extracted using the TIANamp Genomic DNA kit (Tiangen Biotech Co., Ltd., Beijing, China) following the manufacturer’s instructions.

### 2.4. PCR Amplification and Sequencing of ITS-2

Each DNA sample (*n* = 143) was amplified with the universal nematode ITS-2 ribosomal DNA primers (Table S1). PCRs were performed in duplicate in a final volume of 25 μL using 2× Rapid Tag Master mix (Vazyme Biotech, Nanjing, China). The thermal cycling protocol consisted of an initial denaturation at 95 °C for 3 min, followed by 35 cycles of denaturation at 95 °C for 30 s, annealing at 55 °C for 30 s and extension at 72 °C for 1 min, with a final extension at 72 °C for 10 min. PCR products were stored at −20 °C until further processing. A 5 μL aliquot of each PCR product was subjected to electrophoresis on 1.5% agarose gels. Verified PCR products were submitted to Sanger sequencing. The resulting sequences were analyzed using BLAST 2.17.0 to compare with the reference sequences available in GenBank for species-level identification of cyathostomins. The sequence of *Strongylus vulgaris* was included as an outgroup to root the phylogenetic tree. Sequence alignment was performed using the MUSCLE algorithm implemented in MEGA 12. The phylogenetic relationships were inferred using the Maximum Likelihood (ML) method and utilized 1000 bootstrap replicates to assess nodal support. The ML tree with the highest log-likelihood (−2091.66) was selected, and analyses were conducted using MEGA 12. And iTOL (https://itol.embl.de/ (accessed on 1 February 2026)) was used for phylogenetic tree creation.

### 2.5. Benzimidazole Efficacy of Cyathostomins and TBB-ISO-1 Sequencing

FECRT was performed on 11 adult horses, which had not received anthelmintic treatment for at least 5 months prior to the study, under the same husbandry conditions. These horses were orally administered a single dose of albendazole (10 mg/kg.bw) (Hanzhong Xintianyuan Animal Pharmaceutical Co., Ltd., Hanzhong, China). To perform the FECRT, fresh fecal samples were collected from individuals prior to deworming and again from the same individuals 2 weeks post-treatment. Fecal samples were stored at 4 °C and processed within 12 h. The number of eggs in the pre-treatment and post-treatment fecal samples was used to calculate the percent reduction in FEC by the equation: FECR (%) = (EPG _pre_ − EPG _post_)/EPG_pre_ × 100%. FECRT data were analyzed based on an expected efficacy of 99% and a lower efficacy threshold of 90% according to the WAAVP guideline [6].

L3 cyathostomins were cultured from pre-treatment fecal samples of the same 11 horses used in the FECRT, and genomic DNA was extracted for *tbb-iso-1* analysis. PCR analysis was performed as described previously. *tbb-iso-1* primers were designed to target codons 167 (F167Y), 198 (E198A), and 200 (F200Y) (Table S1). PCRs were performed in duplicate in a final volume of 25 μL using 2× Rapid Tag Master mix (Vazyme Biotech, Nanjing, China). The thermal cycling protocol and PCR product process were identical to those used for the ITS-2PCR products of *tbb-iso-1*, which were submitted to Sanger sequencing and analyzed using BLAST. Sequences were aligned in SnapGene 7 to identify homozygous (single peak) and heterozygous (double peak) positions, and allele frequencies were calculated. Genotypic distributions across cyathostomin species were compared in SPSS 27 using chi-square or Fisher’s exact tests [5].

### 2.6. Statistical Analysis

Data were analyzed using the statistical software package SPSS 27 and descriptive statistics (means, median, standard deviations, reduction percentages, etc.). Calculation of the 95% upper and lower confidence limits and interpretation of the findings were as described in Kaplan et al. [6].

## 3. Results and Discussions

### 3.1. Fecal Egg Count

A total of 90 fecal samples were analyzed for intestinal parasite infection. FEC ranged from 0 to 1600 EPG, with a median of 487.5 EPG and a mean ± standard deviation of 549.4 ± 364.4 EPG. Overall, 88.9% (80/90) of samples exceeded the 200 EPG threshold. According to AAEP Parasite Control Guidelines [10], these individuals are classified as moderate-to-high egg shedders. The high prevalence of significant egg shedding observed here is consistent with previous findings in Xinjiang, China [4]. The fecal egg count typically follows the 20/80 rule, which means 20% or less of the horses in one herd excrete 80% of the total egg output. This disparity may result from heavy environmental cyathostomin contamination in the pasture or ineffective parasite control strategies. However, limitations regarding FEC interpretation must be acknowledged. FEC is an unreliable method for estimating the total parasite burden of individual horses, as it fails to detect immature or encysted cyathostomins [16]. Furthermore, FEC cannot morphologically differentiate between large strongyles and cyathostomins.

### 3.2. Cyathostomin Composition

A total of 143 L3 cyathostomins recovered from fecal cultures were subjected to molecular identification using the ITS-2 sequences. All ITS-2 sequences have been deposited in the NCBI database under the corresponding accession numbers: PX289353–PX289472, PX310252–PX310274.

PCR amplification and sequencing successfully assigned the larvae to 9 cyathostomin species, which were *Cylicocyclus nassatus* (*n* = 48; 33.6%), *Cylicostephanus minutus* (*n* = 34; 23.8%), *Cylicostephanus longibursatus* (*n* = 27; 18.9%), *Cyathostomum catinatum* (*n* = 14; 9.8%), *Cyathostomum pateratum* (*n* = 9; 6.3%), *Cylicocyclus leptostomus* (*n* = 5; 3.5%), *Cylicocyclus ashworthi* (*n* = 3; 2.1%), *Cylicostephanus calicatus* (*n* = 2; 1.4%) and *Cylicocyclus insigne* (*n* = 1; 0.7%). The predominance of *Cylicocyclus nassatus* and *Cylicostephanus minutus* in this study aligns with research from other countries [17,18,19]. Differences in the relative abundance of less common species, including *Cyathostomum pateratum* and *Cylicocyclus ashworthi*, may be attributable to regional environmental factors, husbandry practices, and parasite control strategies [16].

**Figure 1:**
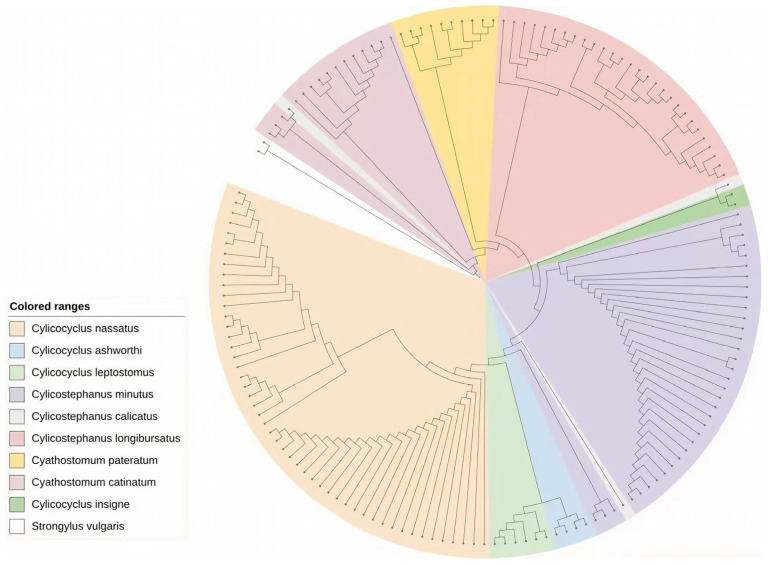
A phylogenetic tree from 143 cyathostomin ITS sequences using the maximum-likelihood method with 1000 bootstrap replicates.

The Maximum Likelihood analysis successfully resolved the genetic relationships among the 143 ITS-2 sequences, clearly demonstrating the presence of nine distinct cyathostomin species (Figure 1). Most species, particularly the dominant species *Cylicocyclus nassatus* and *Cylicostephanus minutus*, formed highly supported monophyletic clades; however, species with limited representation, such as *Cylicostephanus calicatus* (*n* = 2), exhibited significant topological instability in the phylogenetic tree and bootstrap values below 50%. While ITS-2 is efficient for identifying the majority of cyathostomin taxa, its resolution can be insufficient for resolving very closely related or cryptic species. In this study, ITS-2 could not reliably resolve some species, such as *Cylicostephanus calicatus*. This limitation likely explains the low bootstrap support observed for species represented by few sequences. These results indicate that ITS-2-based analysis has limitations and that additional markers, such as COX1, should be used in future studies [20].

### 3.3. Fecal Egg Count Reduction Test with Albendazole

FECRT was conducted on 11 horses to evaluate the efficacy of albendazole. The mean EPG _pre_ was 1277.3 EPG, and the EPG _post_ of 8 horses (72.7%) was 0 EPG. The mean FECRT was 98.8% (95%CI: 97.2–100%) (Table S2). According to the WAAVP guidelines for benzimidazole resistance against cyathostomins, these results indicated that the cyathostomin population in this study remains susceptible to albendazole [10]. This may be attributed to the relatively late widespread adoption of the routine anthelmintics treatment for horses in China. However, three horses were not fully dewormed yet remained below the resistance threshold. It is possible that cyathostomins with slightly reduced sensitivity emerged. Therefore, continued surveillance is warranted to avoid overlooking the development of anthelmintic resistance.

### 3.4. Molecular Analysis of BZ-Resistant Cyathostomin

*Tbb-iso-1* sequences from 55 L3 cyathostomins were successfully obtained. The corresponding *tbb-iso-1* sequences and species identification were provided in Table S2. Among these 55 sequences, codon 153 were all TCC, codon 165 were all TTC, codon 167 were all TCC, codon 172 were all TCA, and codon 200 were all TCC (Table S3). Notably, codon 198 showed variation, including GAG and GAA, which was a synonymous mutation. Overall, no BZ resistance-associated mutations were detected at any of the six loci of *tbb-iso-1*. GAA mutations at codon 198 of *tbb-iso-1* were detected in *Cylicostephanus minutus* (13/55, 23.64%) and *Cylicostephanus longibursatus* (14/55, 25.45%). Similarly, Blackhall et al. also reported a few GAA synonymous mutations in *tbb-iso-1* from a Ukrainian horse herd [21]. Alternative polymorphisms at this locus, such as the E198L variant, have been identified in *Cylicostephanus goldi* [22]. However, although the E198A mutation is a well-established mediator of benzimidazole resistance in ruminants [13], its role in equine cyathostomins remains an area of ongoing investigation. While BZ resistance in cyathostomins has become a global epidemic [23], our findings indicate that the studied horse population remains susceptible in China, which is consistent with a recent study by Jamshidpour in Iran [24]. To our knowledge, the present study represents the first molecular-level evaluation of BZ resistance in cyathostomins within the grazing horse of China.

This study has several limitations that should be acknowledged. Firstly, the study was designed as a population-level baseline survey; therefore, larvae were pooled and not traced back to individual horses. Secondly, the lack of phenotypically resistant isolates prevented the establishment of a definitive causal link between the *tbb-iso-1* codon 200 polymorphism and resistance. Thirdly, due to the high efficacy of albendazole (FECR > 99%), the surviving parasitic load was too low to yield a statistically viable sample size for species differentiation and tubulin genotyping. This technical constraint prevented the analysis of potential “survivors,” which would have been the ideal approach to detect early-stage selection pressure or shifts in species composition.

While finding susceptible genotypes in a phenotypically susceptible population might be expected, the scientific necessity of pre-treatment molecular screening is underscored by the inherent limitations of the FECRT. Phenotypic tests often lack the sensitivity to detect resistant parasites when their proportion in the population remains below 25% [25]. Therefore, molecular screening of pre-treatment larvae was essential to confirm the complete absence of resistant alleles at a sub-clinical level and establish a critical genetic baseline for grazing horses in China. Results from this study are as scientifically vital as detecting resistance for establishing reference baselines for future global surveillance. Furthermore, longitudinal, individual-based pre- and post-treatment fecal sampling is warranted to investigate cyathostomin anthelmintic resistance.

## 4. Conclusions

Overall, the cyathostomin species composition observed in this herd is broadly consistent with reports from other countries, particularly in terms of dominant species. However, differences in the relative abundance of less common species suggest that local management practices, climatic conditions and parasite control strategies may influence the species-level community structure. This herd harbors a diverse community of cyathostomins yet remains susceptible to albendazole. Our findings contribute to a more comprehensive global understanding of parasite population structures and support local veterinary stewardship in preserving current drug efficacy.

## Data Availability

The original contributions presented in this study are included in the article. Further inquiries can be directed to the corresponding authors.

## Supplementary Materials

The following supporting information can be downloaded at: https://www.mdpi.com/article/10.3390/vetsci13020169/s1, Table S1. Sequences of PCR primers. Table S2. Fecal egg count reduction test with albendazole. Table S3. Species identification and sequencing of *tbb-iso-1* SNP position.

## Abbreviations

The following abbreviations are used in this manuscript: AR: anthelmintic resistance; BZ: benzimidazoles; FECR: fecal egg count reduction; FECRT: fecal egg count reduction test; ITS-2: internal transcribed spacer 2; SNPs: single-nucleotide polymorphisms; *tbb-iso-1*: β-tubulin isotype-1 gene.
