# Supplementary material for: Molecular Identification and Benzimidazole Resistance Analysis of Cyathostomins in Chinese Grazing Horses
Source: Vet Sci. 2026 Feb 9;13(2):169. doi: 10.3390/vetsci13020169 (PMC12944989; doi:10.3390/vetsci13020169)
Supplement: Supplementary file 1 [file vetsci-13-00169-s001.zip › vetsci-4021577-supplementary.pdf]

Supplementary Materials

Table S1. Sequences of PCR primers

	Primer	Sequences (5'-3')	Fragment size (bp)
ITS-2	NC1	ACGTCTGGTTCAGGGTTGTT	350/450
	NC2	TTAGTTTCTTTTCCTCCGCT	
Codon 153/165/167	167f	GCTAACTCACTCACTTGGAGGA	120
	167r	CTTTGGTGAGGGAACAAC	
	2f	CAGGGCTTCCAGCTAACTCACTC	130
	167r	CTTTGGTGAGGGAACAAC	
Codon 172/198/200	200f	TGTGGAGCCRTACAATGCT	150
	200r	ACCAAGATGATTCAGATCTCCA	

Table S2. Faecal egg count reduction test with albendazole

Horse	Age (year)	Gender	EPG_{pre}	EPG_{post}	FECRR (%)
1	6	female	75	0	100
2	9	female	175	0	100
3	3	female	275	0	100
4	11	female	275	0	100
5	10	female	600	0	100
6	6	female	750	0	100
7	5	female	1025	0	100
8	18	female	1300	0	100
9	4	male	2025	175	91.4
10	18	male	2075	75	96.4
11	17	male	5475	75	98.6
	Mean±SD		1277.3±1558.4	29.5±56.8	98.8±2.6

Table S3 Species identification and sequencing of *tbb-iso-1* SNP position

Number	153/165	167	172	198	200	Species
1	-	-	-	GAG	TTC	*Cylicocyclus nassatus*
2	TCC	TTC	TCA	GAG/A	TTC	*Cylicostephanus minutus*
3	TCC	TTC	TCA	GAG/A	TTC	*Cylicostephanus minutus*
4	TCC	TTC	TCA	-	-	*Cylicostephanus minutus*
5	TCC	TTC	TCA	GAG/A	TTC	*Cylicostephanus minutus*
6	TCC	TTC	TCA	GAG	TTC	*Cylicocyclus nassatus*
7	-	-	-	GAG/A	TTC	*Cylicostephanus minutus*
8	-	-	-	GAG/A	TTC	*Cylicostephanus minutus*
9	TCC	TTC	TCA	GAG/A	TTC	*Cylicostephanus longibursatus*
10	TCC	TTC	TCA	GAG	TTC	*Cylicocyclus nassatus*
11	-	-	-	GAG/A	TTC	*Cylicostephanus longibursatus*
12	-	-	-	GAG	TTC	*Cylicostephanus longibursatus*
13	-	-	-	GAG	TTC	*Cylicostephanus longibursatus*

14	TCC	TTC	TCA	GAG/A	TTC	*Cylicostephanus longibursatus*
15	TCC	TTC	TCA	GAG	TTC	*Cylicocyclus nassatus*
16	TCC	TTC	TCA	GAG/A	TTC	*Cylicostephanus longibursatus*
17	TCC	TTC	TCA	GAG	TTC	*Cylicostephanus minutus*
18	TCC	TTC	TCA	GAG	TTC	*Cylicocyclus nassatus*
19	TCC	TTC	TCA	GAG	TTC	*Cylicostephanus minutus*
20	TCC	TTC	TCA	GAG/A	TTC	*Cylicostephanus longibursatus*
21	TCC	TTC	TCA	GAG/A	TTC	*Cylicostephanus longibursatus*
22	TCC	TTC	TCA	GAG	TTC	*Cylicostephanus longibursatus*
23	TCC	TTC	TCA	GAG/A	TTC	*Cylicostephanus longibursatus*
24	TCC	TTC	TCA	GAG/A	TTC	*Cylicostephanus minutus*
25	TCC	TTC	TCA	GAA	TTC	*Cylicostephanus minutus*
26	TCC	TTC	TCA	GAG	TTC	*Cylicostephanus longibursatus*
27	TCC	TTC	TCA	GAA	TTC	*Cylicostephanus minutus*
28	-	-	TCA	GAG	TTC	*Cylicostephanus minutus*
29	TCC	TTC	TCA	GAG/A	TTC	*Cylicostephanus longibursatus*
30	TCC	TTC	TCA	GAG/A	TTC	*Cylicostephanus minutus*
31	TCC	TTC	TCA	GAG	TTC	*Cylicostephanus minutus*
32	TCC	TTC	TCA	GAA	TTC	*Cylicostephanus minutus*
33	TCC	TTC	TCA	GAG	TTC	*Cylicocyclus nassatus*
34	TCC	TTC	TCA	-	-	*Cylicostephanus calicatus*
35	-	-	-	GAG	TTC	*Cylicostephanus longibursatus*
36	-	-	-	GAA	TTC	*Cylicostephanus longibursatus*
37	-	-		GAG/A	TTC	*Cylicostephanus minutus*
38	-	-		GAG	TTC	*Cylicostephanus minutus*
39	-	-		GAG	TTC	*Cylicostephanus minutus*
40	-	-		GAG/A	TTC	*Cylicostephanus longibursatus*
41	TCC	TTC		GAG/A	TTC	*Cylicostephanus longibursatus*
42	TCC	TTC		GAG	TTC	*Cylicostephanus longibursatus*
43	TCC	TCC		GAG/A	TTC	*Cylicostephanus minutus*
44	-	-		GAG	TTC	*Cylicostephanus minutus*
45	TCC	TTC		GAG	TTC	*Cylicostephanus minutus*
46	TCC	TTC		GAG/A	TTC	*Cylicostephanus minutus*
47	TCC	TTC		-	-	*Cylicostephanus minutus*
48	TCC	TTC		GAG	TTC	*Cylicostephanus longibursatus*
49	TCC	TTC		GAA	TTC	*Cylicostephanus longibursatus*
50	-	-		GAA	TTC	*Cylicostephanus longibursatus*
51	-	-		GAG	TTC	*Cylicostephanus minutus*
52	-	-		GAG	TTC	*Cylicostephanus minutus*
53	TCC	TTC		GAA	TTC	*Cylicostephanus longibursatus*
54	TCC	TTC		GAG	TTC	*Cylicostephanus minutus*
55	TCC	TTC		GAG	TTC	*Cylicocyclus nassatus*

"–"denotes sequencing failure.
